# Genome-Driven Functional Validation of *Bacillus amyloliquefaciens* Strain MEPW12: A Multifunctional Endophyte for Sustainable Sweet Potato Cultivation

**DOI:** 10.3390/microorganisms13061322

**Published:** 2025-06-06

**Authors:** Yiming Wang, Jingwen Hao, Jingsheng Gu, Jiaying Wu, Yongjing Zhang, Ting Liang, Haimeng Bai, Qinghe Cao, Jihong Jiang, Ludan Li, Xiaoying Cao

**Affiliations:** 1 The Key Laboratory of Biotechnology for Medicinal and Edible Plants of Jiangsu Province, School of Life Sciences, Jiangsu Normal University, Xuzhou 221116, China; 1020210013@jsnu.edu.cn (Y.W.); gujingsheng@jsnu.edu.cn (J.G.);; 2 Xuzhou Institute of Agricultural Sciences in Jiangsu Xuhuai District, Xuzhou 221131, China

**Keywords:** endophyte, sweet potato, growth promoting bacteria, genome, pan-genomic analysis

## Abstract

Sweet potato (*Ipomoea batatas* (L.) Lam.), as an important crop, is rich in polyphenols, vitamins, minerals, and other nutrients in its roots and leaves and is gradually gaining popularity. The use of endophytic bacteria to improve the quality of sweet potato can protect the environment and effectively promote the sustainable development of the sweet potato industry. In this study, 12 strains of endophytic bacteria were isolated from sweet potato. Through nitrogen fixation, phosphorus solubilization, indoleacetic acid production, siderophore production, ACC deaminase production, and carboxymethyl cellulose production, three strains with multiple biological activities were screened out. Among them, MEPW12 had the most plant growth-promoting functions. In addition, MEPW12 promoted host chlorophyll accumulation and inhibited pathogen growth and colonization in sweet potato roots and can utilize various carbon sources and salts for growth. It can also grow in extreme environments of high salt and weak acid. MEPW12 was identified as *Bacillus amyloliquefaciens* with a genome size of 3,928,046 bp and a GC content of 46.59%. After the annotation of multiple databases, it was found that MEPW12 had multiple enzymatic activities and metabolic potential. Comparative genomics and pan-genomics analyses revealed that other *Bacillus* sp. strains of MEPW12 have similar functions. However, due to adaptation to different growth environments, there are still genomic differences and changes. Inoculation with MEPW12 induced the high expression of *IbGH3.10*, *IbERF1*, and other genes, thereby promoting the growth of sweet potatoes. *Bacillus amyloliquefaciens* strain MEPW12 is a sweet potato endophyte with multiple growth-promoting functions, which can promote the growth of sweet potato seedlings. This study provides new microbial resources for developing microbial agents and improving the quality of sweet potatoes.

## 1. Introduction

With the increasing human demand for food and crops, chemical fertilizers are widely used to promote crop growth, resulting in a series of environmental hazards, such as soil pollution, soil fertility decline, and the presence of harmful residues in food [1,2]. Therefore, it is necessary to find a safe and sustainable way to replace the application of chemical fertilizers to improve crop quality and reduce environmental pollution.

Endophytes are microbial groups that are widely present in healthy plant tissues and coexist harmoniously with the host plant without causing major damage to the host [3]. Endophytes can play an important role in influencing the quality and yield of the host through specialized microbe–plant interactions [4,5]. Many studies have revealed beneficial relationships between endophytes and their hosts to promote plant growth, such as nitrogen fixation, phosphorus solubilization, production of 1-aminocyclopropane-1-carboxylic acid (ACC) deaminase, production of plant hormones such as indole-3-acetic acid (IAA), secretion of siderophores, and production of carboxymethyl cellulose (CMC) cellulase [6,7,8,9]. Endophytes can also produce volatile organic compounds (VOCs) to help hosts resist biotic and abiotic stresses, enhance nutrient uptake, and improve the soil environment [10,11]. At the same time, the researchers demonstrated that endophytes can regulate the accumulation and production of active ingredients in plants and can produce active products that are the same or similar to the active ingredients in the host to increase the active ingredients in the host [3,12,13,14,15]. Endophytes have gradually become a research hotspot in sustainable agricultural development due to their various activities and functions. Screening endophytes to promote crop growth and preparing biological agents will greatly improve agricultural ecology.

Sweet potato (*Ipomoea batatas* (L.) Lam.) has become increasingly popular due to its rich nutritional value and health benefits. Sweet potatoes are rich in polyphenols, vitamins, and minerals and have multiple health benefits such as enhancing immunity, lowering blood sugar, preventing night blindness, and preventing atherosclerosis [16,17]. The cultivation of sweet potatoes is widely practiced throughout the world and plays a crucial role in alleviating famine in developing countries [18]. How to improve the yield and nutrient content of sweet potatoes without causing environmental pollution in the production process is very important for the sweet potato industry.

Bacterial whole genome sequencing refers to sequencing the whole genome of bacteria to obtain the complete DNA sequence information, which is mainly used for bacterial species identification, gene function research, drug target discovery, and drug resistance analysis [19]. Through sequence analysis and functional gene annotation, the phenotypic mechanism of strains can be understood at the gene level [20]. The exploration of plant growth promotion mechanisms by microbial genome analysis has mainly focused on the identification of genes related to key functions such as nutrient acquisition, hormone production, stress resistance, and the prediction of potential interaction mechanisms between bacteria and their hosts based on genomic data will help in the development of biofertilizers [21]. Comparative genomics and pan-genomics complement each other. The former focuses on the differences and evolutionary paths among strains, and the latter comprehensively describes the diversity of gene pools of species. They jointly promote the understanding of bacterial genetic mechanisms, the development of biological resources, and the advancement of agricultural applications and are important methods in microbiology research [22,23].

In this study, 12 endophytic bacteria were isolated and cultured from sweet potatoes, and strains with various biological activities were selected. In addition, the effects of strains on the growth of sweet potatoes and the potential mechanism were explored by the inoculation of sweet potatoes, and the potential functions of strains were analyzed by genome sequencing. Comparative genomics and pan-genomics were used to provide a theoretical basis for the function mining of plant pro-growth bacteria and reveal the complex association of gene–function–environment. We hope to explore the potential functions, evolution, and environmental adaptability of endophytic bacteria in sweet potatoes through functional verification, genomic analysis, comparative genomics, and pan-genomics, aiming to provide new microbial resources for the sweet potato industry, sustainable agricultural development, and soil environmental protection.

## 2. Materials and Methods

### 2.1. Sampling and Isolation of Bacterial Endophytes

The sweet potato seedlings (3-month-old, Xuzishu8) were collected from Xuzhou Academy of Agricultural Sciences (Xuzhou, China). Disease-free, pest-free, and uniform sweet potato seedlings were carefully selected and transplanted at Jiangsu Normal University (Xuzhou, China). The plant material was surface-sterilized with 5% sodium hypochlorite for 2 min, transferred to 70% ethanol solution for 2 min, rinsed twice with sterile distilled water, chopped, and then ground. Approximately 2 g of sweet potato leaves were spread into the isolation medium (R2A agar and Nutritional agar) and incubated for 48 h at 37 °C [24]. Single colonies were picked and streaked into LB medium.

### 2.2. Functional Identification of Endophytic Bacteria

#### 2.2.1. Detection of Phosphorus Solubilization

The modified Pikovskaya (PVK) solid medium was used to evaluate the ability of the strains to dissolve organophosphorus [25]. The inoculated strains were stored at 28 °C for 7 days. The presence of a transparent halo around the strain indicates that the strain is phosphate-solubilizing. The experiment for phosphorus solubilization was repeated three times.

#### 2.2.2. Detection of Nitrogen Fixation

The strains were inoculated onto nitrogen-free agar medium and then incubated at 28 °C [26]. If the strain can still grow vigorously after three generations of culture, it indicates that the strain has nitrogen fixation activity. Three consecutive rounds of culture verification were conducted using nitrogen-free agar medium, and the growth status of the strains in the last round was observed.

#### 2.2.3. Detection of Siderophore Production

Siderophore production was assessed using CAS agar plates, and the appearance of yellow halos indicated siderophore production [27]. Siderophore production experiments were repeated three times.

#### 2.2.4. Detection of 1-Aminocyclopropane-1-Carboxylic Acid (ACC) Deaminase Production

The activity of ACC deaminase was detected by Dworkin and Foster medium, which utilizes ACC as a sole source of nitrogen [28]. To enable strains to utilize 3 mM ACC as the sole nitrogen source, strains were inoculated and incubated at 28 °C for 7 days. The strains were continuously cultured for 3 rounds; 7 days after the last round of culture, the growth of the strains was observed.

#### 2.2.5. Detection of Cellulase Production

Cellulase activity of the strains was measured using microsalt CMC medium. The strains were inoculated into Petri dishes and incubated at 28 °C for 7 days. The plates were then stained with 0.5% Congo red solution for ten minutes and then washed with 9% (*w*/*v*) sodium chloride solution [29]. The formation of a transparent circle around the colony was identified as having cellulase activity.

#### 2.2.6. Detection of Indole-3-Acetic Acid (IAA) Production by Strains

IAA production activity of the strains was determined by the Salkowski reagent method [30]. The activated strains were inoculated into the Gaussian liquid medium containing 0.5 g/L tryptophan and incubated at 28 °C for 3 days at a shaking rate of 150 r/min to obtain fermentation broth. The bacterial suspension was centrifuged, the supernatant was collected, filtered, and sterilized, and the chromogen was added and incubated at 25 °C for 30 min. The absorbance of OD530 nm was measured after being placed in a 96-well plate. The experiment was repeated three times. At the same time, 10 mg of IAA was dissolved in 100 mL of water, and the standard curve was prepared after gradient dilution to quantify IAA production.

### 2.3. Plant Growth Promotion Experiment

The strain on the LB solid was inoculated in 200 mL of LB liquid medium, and after 3 days of culture, the bacterial solution was centrifuged at 10,000 r/min for 5 min, and the supernatant was discarded. The strain was resuspended using sterile water, and the concentration was adjusted to OD600 = 1 using a UV spectrophotometer for use [31]. Sweet potato seedlings (3-month-old) with similar morphology were cut off, weighed one by one and the initial weight (W_0_) recorded, planted in sterilized soil with a mixture of sand and vermiculite (2:1) to prepare pot (10 cm × 10 cm) plants, and allowed to grow for one week. Then, 50 mL of bacterial suspension was inoculated into the rhizosphere soil of sweet potato seedlings by root irrigation twice on the first and third days, and only watered sweet potato seedlings were set as the control group. After 7 days, the sweet potato seedlings were carefully dug out, the soil on the roots was washed with water, and the surface water was sucked off with filter paper. The length of the longest three roots was measured, and the fresh weight of each sweet potato seedling was measured (W) [32]. The growth rate of sweet potato seedlings was calculated according to the formula [(W − W_0_) /W_0_] × 100%.

### 2.4. Determination of the Chlorophyll Content

The contents of chlorophyll a and chlorophyll b were measured. A third expanded leaf was taken from the tip down, ground in 95% ethanol, and sonicated for 30 min. Subsequently, the absorbance values at 663 nm and 646 nm were determined, and the concentration was calculated according to the method of Xu et al. and the Porra formula [33,34], with three replicates.

### 2.5. Identification of Growth Promoting Bacteria

Selected bacterial isolates were identified by 16S rRNA gene analysis, and the genomic DNA of the purified strains was extracted using FastPure Bacteria DNA Isolation Mini Kit-BOX 2 (Vazyme Biotech Co., Ltd., Nanjing, China). The 16S rRNA gene was amplified using the universal bacterial primers 27F (5′-AGAGTTTGATTCTGGCTCAG-3′) and 1492R (5′-GGTTACCTTGTTACGACTT-3′). PCR was performed according to the following procedure: initial denaturation at 94 °C for 5 min. This was followed by 35 cycles at 94 °C for 1 min, 57 °C for 30 SEC, 72 °C for 30 SEC, and a final extension step lasting 7 min at 72 °C. The enzyme used in the PCR reaction was from the NaviScript^®^ Rapid PCR Master Mix (Synomebio Co., Ltd., Shanghai, China). The obtained PCR products were sent to Sangon Biotech Co., Ltd. (Shanghai, China) for testing. The sequences were compared and analyzed with those in the GenBank database using the BLAST alignment tool (http://www.ncbi.nlm.nih.gov/blast/, accessed on 24 July 2024). The species of the isolates were identified as the highest-scoring species in the BLAST results. Combining 16S rDNA sequences from other strains, a phylogenetic tree was constructed using the maximum likelihood method with MEGA 11, and 1000 bootstrap replicates were executed to assess the tree’s robustness.

### 2.6. Physiological and Biochemical Identification of MEPW12

The utilization and identification experiments of anaerobic growth, Voges–Proskauer (V–P), citrate, propionate, D-xylose, L-arabinose, D-mannitol, gelatin liquefaction, growth with 7% sodium chloride, growth with PH5.7, nitrate reduction, and starch hydrolysis were carried out using biochemical identification strips (HBIG14, Hopebio, Qingdao, China) [35].

### 2.7. Antagonistic Activity of MEPW12 Against Sweet Potato Pathogens

The antagonistic activity of strain MEPW12 was determined using two pathogenic fungi associated with sweet potatoes. They were *Ceratocystis fimbriata* BMPZ13 (Black rot disease) and *Fusarium solani* (Mart) sacc. f. sp. batatas MeClure (Root rot disease), and the pathogens were stored at Jiangsu Normal University. Detection was performed using the in vitro dual culture assay method, in which strains were grown on PDA plates for 5 days, and repeated three times [35]. The diameter of the fungal colony was recorded daily to calculate the growth inhibition rate according to the following formula: inhibition rate (%) = (diameter of the control fungal colony-diameter of the fungal colony in the presence of an endophytic strain)/(diameter of the control fungal colony) × 100.

### 2.8. Detection of MEPW12 Colonization

The colonization of MEPW12 was determined by quantitative real-time PCR (qPCR) [36,37]. The specific primers for housekeeping gene *gyrA* were designed according to the method of Liu et al. (*gyrA3*-F: 5′-GCDGCHGCNATGCGTTAYAC-3′ and *gyrA3-R*: 5′-ACAAGMTCWGCKATTTTTTC-3′) [38]. The roots of sweet potato seedlings in the experimental group and the control group were collected. Surface disinfection was performed using the method described in step 2.1 after rinsing with running water for 30 s. The DNA of the samples was extracted by using FastPure Bacteria DNA Isolation Mini Kit-BOX 2 (Vazyme Biotech Co., Ltd., Nanjing, China). The qRT-PCR analysis was completed using the NaviScript^®^ Pro SYBR Green qPCR Master Mix-Blue (Synomebio Co., Ltd., Shanghai, China) and StepOne Plus real-time PCR System (ABI, Los Angeles, CA, USA). The relative transcript levels were calculated using the 2^−ΔΔCt^ method, and the average cycle threshold (Ct) was normalized to that of *ACTIN* [35]. The cycling program was as follows: 95 °C for 30 s, followed by 45 cycles of 95 °C for 10 s and 60 °C for 30 s. All qRT-PCR tests were repeated three times.

### 2.9. Whole Genome Analysis of Strain MEPW12

The whole genome sequencing was completed by the BENAGEN Company (Wuhan, China) using the third-generation Nanopore sequencer and the second-generation Illumina HiSeq technology. Freely available software Unicycler (0.4.8) (https://github.com/rrwick/Unicycler, accessed on 1 December 2024) was used to assemble the obtained data [39]. The coding genes of the assembled genome were predicted by the Prokka software (1.1.2), and the predicted gene sequences were compared with GO, KEGG, CAZy, Pfam, COG, and other functional databases by BLAST to obtain the gene function annotation results. The online software AntiSMASH (https://antismash.secondarymetabolites.org, accessed on 1 December 2024) was used to predict the secondary metabolite synthesis genome cluster in MEPW12 and analyze the possible metabolites [40].

### 2.10. Comparative Genomics and Pan-Genomic Analysis

After sequence alignment, the complete genomes of four model *Bacillus* strains, UCMB5036, DSM7, FZB42, and SQR9, were downloaded from NCBI and analyzed for collinearity analysis using Mauve software (3.9.x) and for ANI analysis using the IPGA (https://nmdc.cn/ipga/, accessed on 1 December 2024) online tool [41].

Pan-genome analysis of five *Bacillus* sp. strains was performed using the IPGA online tool. Quality control of genomic sequences was performed first to ensure that sequences with a completeness greater than 90% and a contamination rate less than 5% were analyzed. PANOCT, OrthoMCL, Roary, panX, OrthoFinder, Panaroo, and PPanGGoLiN modules were selected for analysis, and the identity, Ratio (core), and Support parameters were set as 70, 0.95, and −1, respectively [42].

### 2.11. RNA Extraction and Quantitative Reverse Transcription PCR (qRT-PCR) Validation

Collect leaves of sweet potato seedlings. The total RNA of the leaf samples of sweet potatoes was extracted using an RNA Pure Plant Kit (Vazyme Biotech Co., Ltd., Nanjing, China) according to the instructions and reverse transcribed to cDNA using the NaviScript^®^ RT Master Mix with a gDNA Sweeper (Synomebio Co., Ltd., Shanghia, China). Specific qPCR primers for key genes were designed based on previous transcriptome data (Accession number: PRJNA1126169) from sweet potato leaves (Supplementary Table S1), and qRT-PCR analysis is consistent with step 2.7.

### 2.12. Data Analysis

The graphics are drawn using GraphPad Prism software (v8.0.2.263), and the tables are drawn using Excel. Single-factor analysis of variance (ANOVA) was used to evaluate the differences in the data obtained from the experiment, with a significance level set at *p* < 0.05. All experiments were repeated at least three times.

## 3. Results

### 3.1. Isolation and Functional Identification of Endophytes from Sweet Potatoes

Aerial parts of sweet potatoes (Xuzishu8) were collected from Jiangsu Normal University (Xuzhou, China), and endophytic bacteria were isolated and cultured in LB medium from selected colonies. After morphological observation and duplication removal, a total of 12 culturable endophytic bacteria were obtained, provisionally designated as MEPW1 to MEPW12 (Supplementary Figure S1).

**Figure 1:**
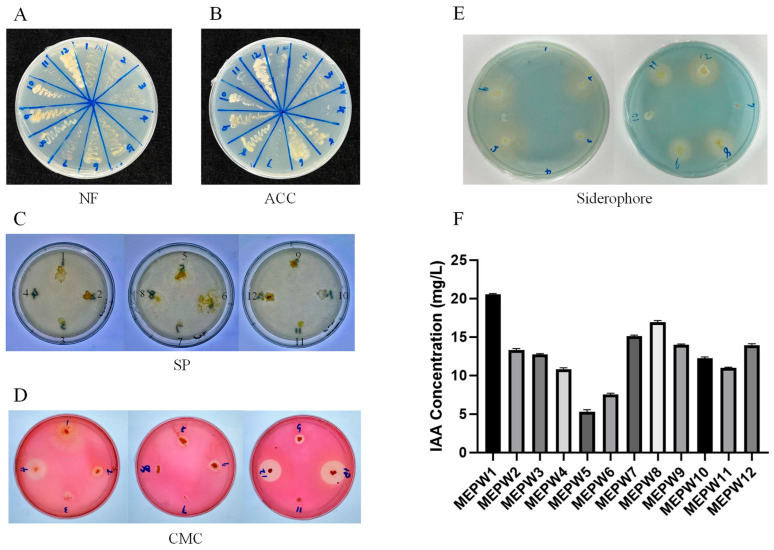
Functional identification of endophytic bacteria. (**A**) Nitrogen fixation capacity testing; (**B**) ACC deaminase detection; (**C**) Solubilization of phosphate testing; (**D**) Detection of CMC cellulase production capacity; (**E**) Siderophore production capacity testing; (**F**) IAA quantification.

As the results show below (Figure 1), all bacteria except MEPW4 and MEPW11 had the function of nitrogen fixation (Figure 1A). MEPW2, MEPW3, MEPW5, MEPW6, MEPW8, MEPW9, MEPW10, MEPW11, and MEPW12 have the activity to produce ACC deaminase (Figure 1B). In the phosphate solubilizing ability test, only MEPW12 showed a faint transparent circle, while other strains did not show phosphate-solubilizing activity (Figure 1C). MEPW4, MEPW5, MEPW6, MEPW9, MEPW10, and MEPW12 had the activity of CMC cellulase production, and the transparent circle area of MEPW4, MEPW10, and MEPW12 was larger, indicating their stronger activity (Figure 1D). Siderophore production assays conducted on these isolates revealed that strains including MEWP2, MEPW3, MEPW5, MEPW6, MEPW8, MEPW9, MEPW11, and MEPW12 displayed significant production capacity (Figure 1E). Surprisingly, most of the endophytes, especially MEPW1, MEPW7, and MEPW8, possessed indole acetic acid production capacity (Figure 1F).

By counting the functions of all strains (Table 1), we found that MEPW12 possessed all the functions in the assay. Although MEPW1, MEPW7, and MEPW8 showed high IAA production activity, they exhibited limited functionality in other assays. MEPW6 and MEPW9 were more active than other strains, except for their phosphate-solubilizing function. Therefore, MEPW6, MEPW9, and MEPW12 were selected for subsequent study.

**Figure 2:**
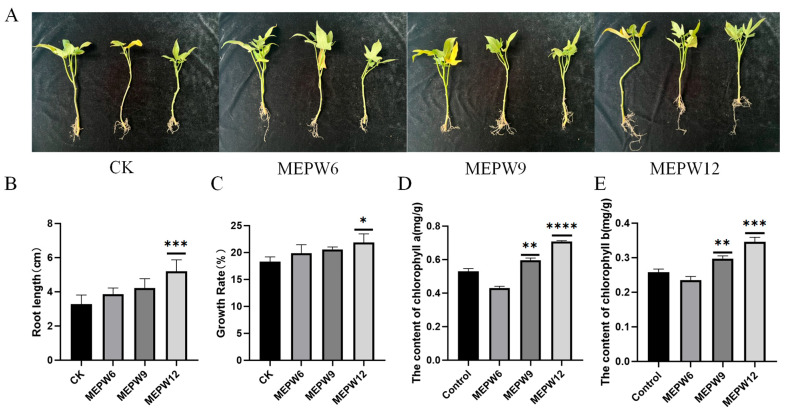
Effect of endophytes on the growth of sweet potato seedlings. (**A**) Growth appearance of sweet potato seedlings inoculated with different strains; (**B**) The effects of endophytes on the root length of sweet potato seedlings; (**C**) Effects of endophytes on the weight of sweet potato seedlings. (**D**) Chlorophyll a contents of leaves; (**E**) Chlorophyll b contents of leaves; Significance levels: * *p* < 0.05, ** *p* < 0.01, *** *p* < 0.001, **** *p* < 0.0001.

After inoculating these three strains of bacteria into sweet potato seedlings, we found that the growth rate of root length and fresh weight of sweet potato seedlings treated with MEPW12 was significantly improved. However, the growth rate of sweet potato seedlings treated with MEPW6 and MEPW9 was not significant (Figure 2A–C). Sweet potato seedlings inoculated with MEPW9 and MEPW12 showed significant increases in chlorophyll a and chlorophyll b, with MEPW12 showing the most significant improvement (Figure 2D,E). The above experiments indicated that MEPW12 had a stronger plant growth-promoting function and was therefore selected for subsequent studies.

### 3.2. Molecular Identification of MEPW12 and Identification of Other Physiological and Biochemical Functions

**Figure 3:**
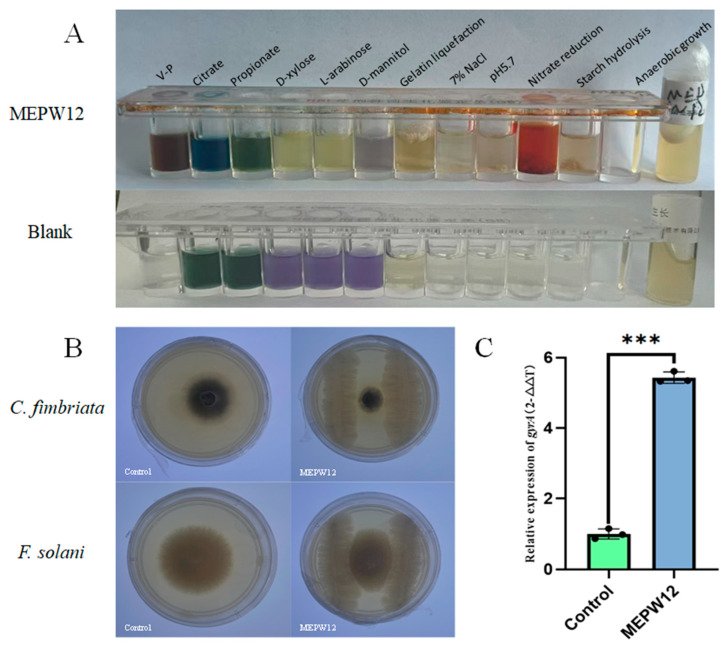
Identification of physiological and biochemical functions of MEPW12, and detection of antibacterial and colonization ability. (**A**) Physiological and biochemical analysis of MEPW12; (**B**) The inhibitory effect of MEPW12 on sweet potato pathogens; (**C**) Expression of *gyrA* in the samples; Significance levels: *** *p* < 0.001.

By physiological and biochemical identification, the V–P test of MEPW12 was positive, indicating that MEPW12 could ferment glucose to pyruvate. Meanwhile, MEPW12 could grow with citrate, D-xylose, and L-arabinose. MEPW12 could grow in the presence of 5.7% NaCl and pH5.7, indicating its tolerance to high salt and a weak acid environment. The positive results of gelatin liquefaction and starch reduction experiments indicated that MEPW12 also had the ability to produce gelatinase and amylase (Table 2, Figure 3A).

In addition to promoting plant growth, MEPW12 showed antagonistic activity against two pathogens of sweet potatoes. The average inhibition rates of MEPW12 against *C. fimbriata* and *F. solani* were 60.87% and 57.47%, respectively (Figure 3B). Moreover, the expression of *gyrA* was significantly higher after inoculation with MEPW12 than that in the control group, indicating that MEPW12 could colonize the roots of sweet potato seedlings after inoculation with root irrigation (Figure 3C).

**Figure 4:**
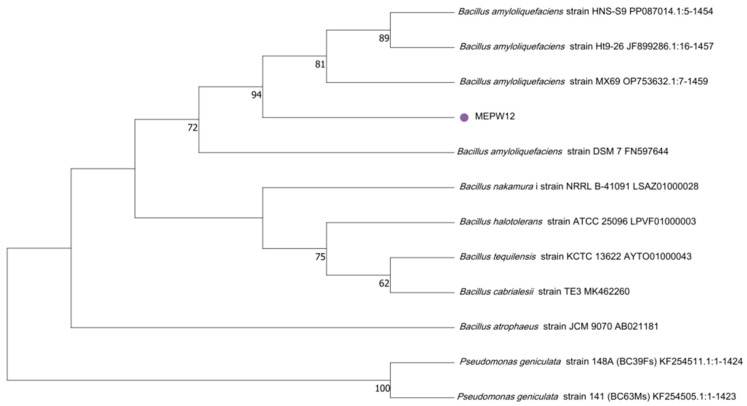
Phylogenetic tree of MEPW12 constructed based on the maximum likelihood method of MEGA 11.

The genomic DNA of MEPW12 was amplified using universal primers 27F and 1492r, and the rDNA gene sequences of 1453bp were amplified by PCR. The NCBI database was used for BLAST sequence alignment, the highest similarity and relatively high sequences were selected for subsequent analysis, the candidate sequences were downloaded, and the sequence alignment was performed using MEGA11. MEPW12 could be identified as *Bacillus amyloliquefaciens*, so it was tentatively named *Bacillus amyloliquefaciens* strain MEPW12 (GenBank: PQ533237.1) (Figure 4).

### 3.3. Genomic, Comparative Genomic, and Pan-Genomic Analyses of MEPW12

**Figure 5:**
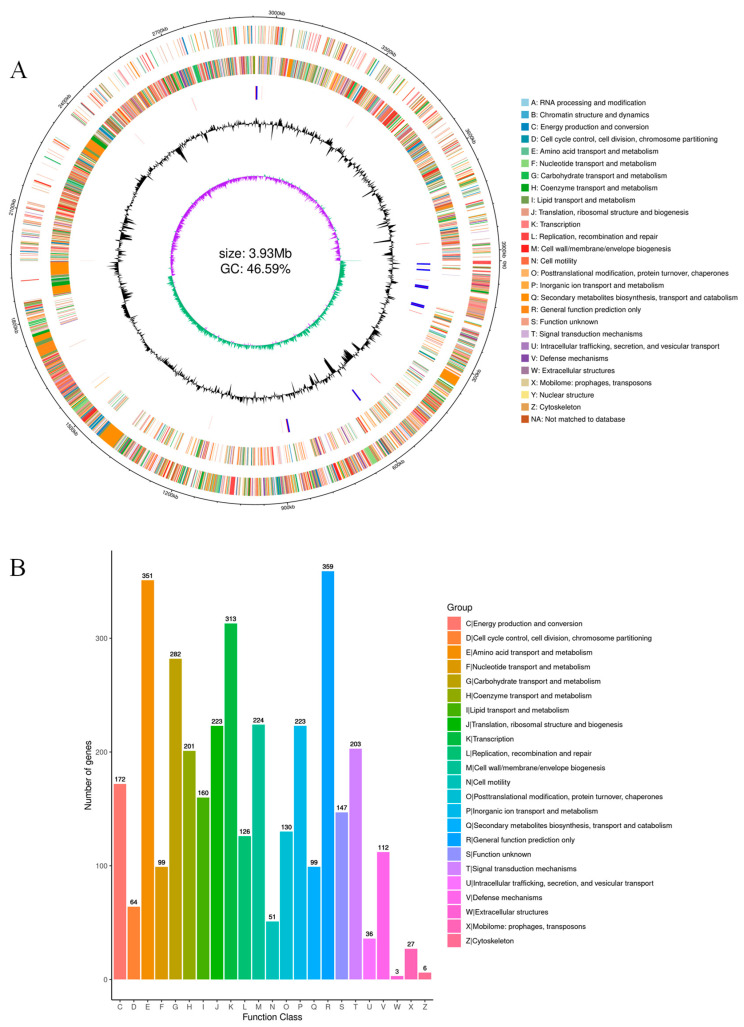
Genome circle mapping and COG annotation of MEPW12. (**A**) Genomic circle diagram of strain MEPW12; (**B**) COG database annotations for strain MEPW12.

The results of sequencing showed that strain MEPW12 contained a chromosome of 3,928,046 bp with an average GC content of 46.59%, which was annotated to 3752 coding genes with a total length of 3,471,636 bp and an average length of 925 bp, accounting for 88.38% of the whole genome (Figure 5A). Genes involved in IAA production, siderophore production, nitrogen generation, phosphate solubilization, and uptake were screened by gene annotation (Table 3), which was consistent with the previous functional identification of MEPW12.

A total of 3057 functional genes were annotated in the COG database of strain MEPW12 (Figure 5B), of which general function prediction only was the most annotated (359), followed by amino acid transport and metabolism (351), transcription (313), carbohydrate transport, and metabolism (282).

**Figure 6:**
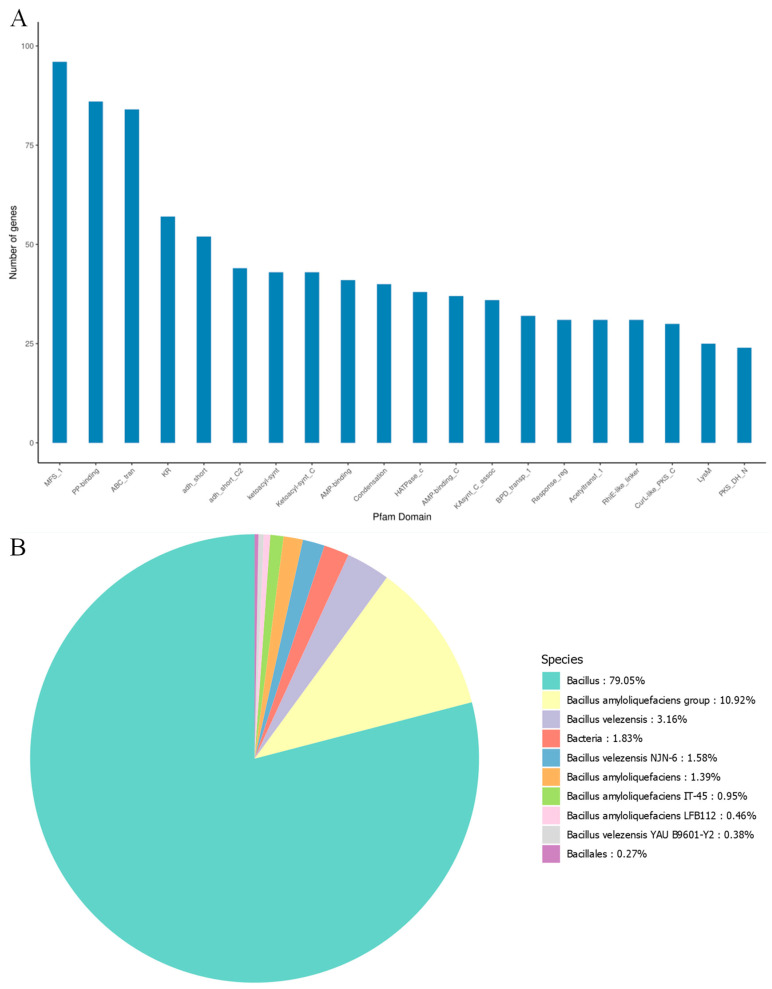
Pfam database annotations and NR database annotations for MEPW12. (**A**) Pfam database annotations for strain MEPW12; (**B**) NR database annotations for strain MEPW12.

The Pfam database provides a relatively complete and accurate classification of protein families and functional domains. In the analysis of strain MEPW12, 3348 genes with biological significance were annotated in the Pfam database, indicating that the genome of strain MEPW12 contains rich functional information and has important biological value (Figure 6A). Strain MEPW12 annotated 3749 genes in the NR database (Figure 6B), among which 79.05% of genes were annotated in *Bacillus* sp., accounting for the highest proportion, followed by *Bacillus amyloliquefaciens*, accounting for 10.92%. The carbohydrate enzymes of strain MEPW12 were predicted by the CAZy database, and 105 genes were annotated. Among them, glycoside hydrolases were annotated the most, with 40 genes, followed by glycosyltransferases and carbohydrate esterases, with 35 and 17 genes, respectively (Table 4).

**Figure 7:**
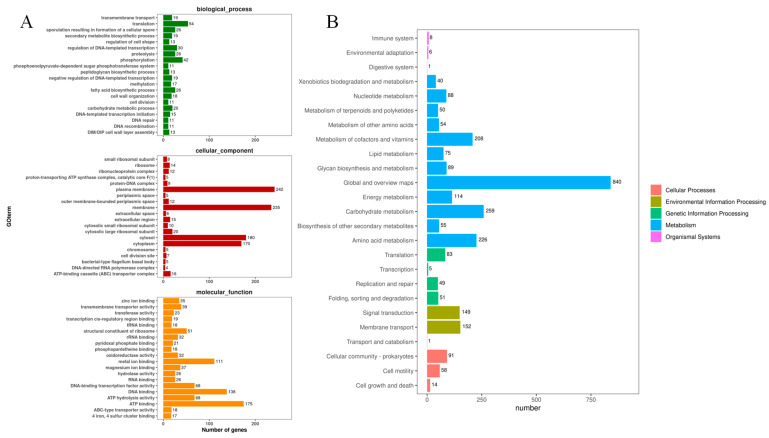
GO functional annotation and KEGG signaling pathways annotation for MEPW12 (**A**) GO functional annotation map of strain MEPW12; (**B**) Results of KEGG signal pathway enrichment analysis of strain MEPW12.

GO (Gene Ontology) annotation information is simplified to obtain the classification of GOslim, and the functions of genes are summarized and counted from three aspects: cell composition, molecular function, and biological process. The top 20 secondary classifications of GOslim with the most annotations under each classification are selected for drawing. In molecular functions, ATP binding has the largest number of genes, with 175, followed by DNA binding and metal binding, with 138 and 111, respectively. The number of genes enriched by translation and phosphorylation in biological processes is relatively large, with 54 and 42 genes, respectively. Cellular components have the largest number of genes related to the plasma membrane, with 242 genes, followed by the membrane (235), cytosol (180), and cytoplasm (170) (Figure 7A).

A total of 2935 genes were enriched in the KEGG signaling pathway. There are 2751 genes enriched in the four first-order signal pathways, namely, environmental information processing, cellular processes, genetic information processing, and metabolism, among which the number of genes related to metabolic pathways is the largest, with 2098. Among them, the global overview map, carbohydrate metabolism, amino acid metabolism, and metabolism of co-factors and vitamins are the main metabolic pathways of strain MEPW12 (Figure 7B).

The antiSMASH online database predicts that there are 13 secondary metabolite gene regions in the MEPW12 genome, among which 6 gene regions show 100% similarity with macrolactone H, bacillarene, fengycin, difficidin, bacillibactin, and bacilysin. Region4 encodes a PKS-like gene cluster, which is only 7% similar to the known gene cluster butirosin A/butirosin B. In addition, the most similar gene clusters were not detected for the three regions 5, 9, and 10 encoding terpene (from 1,048,459 to 1,069,199), terpene (from 2,001,477 to 2,023,360), and T3PKS (Table 5). This suggests that MEPW12 has a strong potential to produce secondary metabolites, especially antibiotics, and antagonize pathogens in this way.

**Figure 8:**
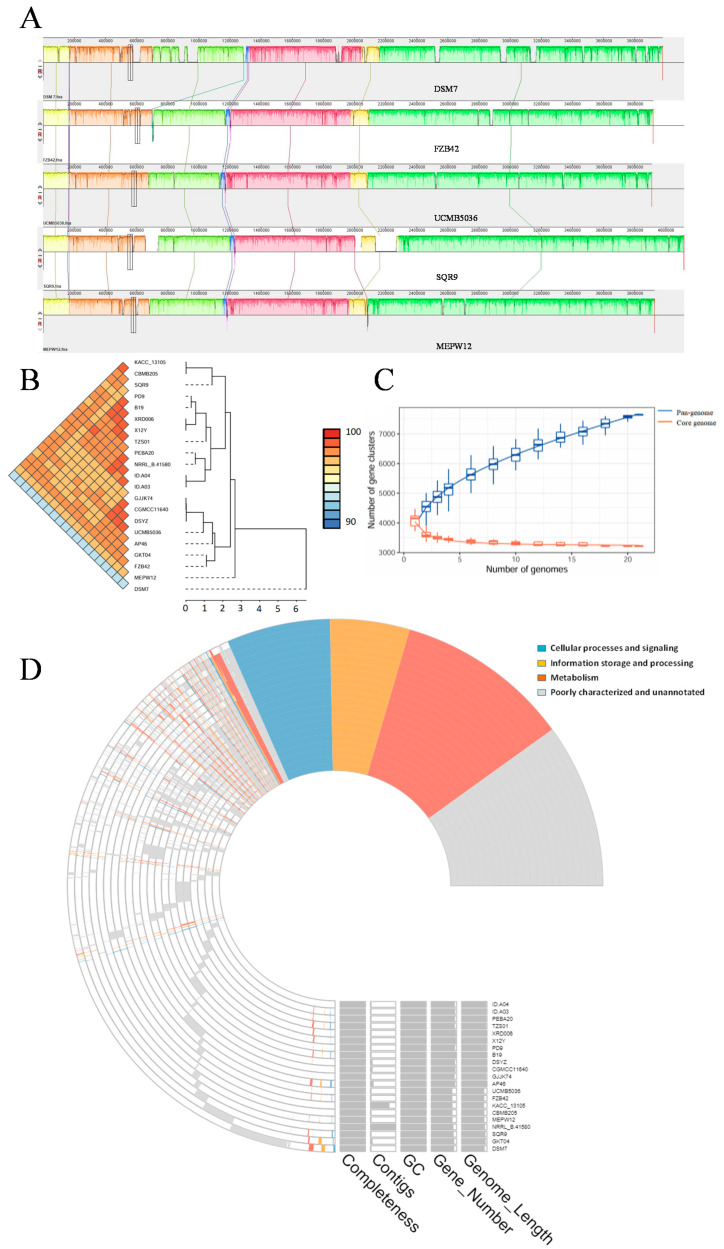
Comparative genomics and pan-genomic analysis. (**A**) Collinearity analysis; (**B**) ANI analysis; (**C**) Pan-genome, core genome, and gene cluster relationship chart; (**D**) Pan-genome map based on COG annotation.

We downloaded 20 *Bacillus* sp. genomes from NCBI, and 4 were randomly selected for collinearity analysis with MEPW12. The results showed that the nucleotide sequences of five strains were highly similar, but there were also genomic rearrangements such as turnover and translocation (Figure 8A). ANI analysis of these 21 Bacillus strains revealed genomic similarity greater than 97% between most strains, with the exception of DSM7 (all greater than 93%), indicating that the 21 strains had a strong similarity and were the same species (Figure 8B). Pan-genome analysis of 21 *Bacillus* strains showed that the number of pan-genomes increased to 7645, and the number of core genomes decreased to 3217 and gradually stabilized, indicating that the pan-genome of *Bacillus* sp. was open and could acquire new genetic information from different environments (Figure 8C). The COG-annotated pan-genome map showed that of the 3217 core genes, 1152, 535, and 694 genes were used in metabolism, information storage and processing, and cellular processes, and 836 genes were poorly characterized and unannotated. Strain DSM7 has the most unique genes (744 genes), while strain UCMB5036 has the smallest genome with only 20 unique genes. Strain MEPW12 possesses 231 unique genes, including 38 metabolism, 23 information storage and processing, and 12 cellular process and signaling genes (Figure 8D).

### 3.4. Effect of MEPW12 on the Expression of Growth-Related Genes in Sweet Potatoes

**Figure 9:**
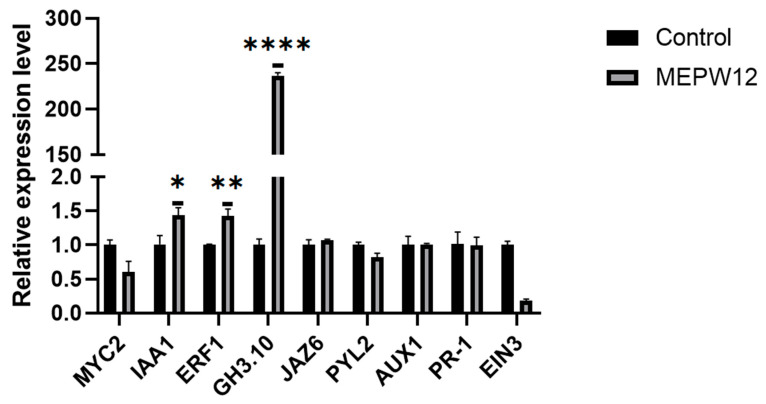
Changes in gene expression involved in the plant hormone signaling pathway. Significance levels: * *p* < 0.05,** *p* < 0.01, **** *p* < 0.0001.

To further explore the mechanism by which MEPW12 promotes the growth of sweet potato seedlings, we extracted RNA from the MEPW12 group and the control group. According to our previous transcriptome test of sweet potatoes, the genes related to plant hormone signal transduction were selected to design primers for RT-qPCR (Supplementary Table S1). The results showed that MEPW12 inoculation induced the high expression of *IbIAA1*, *IbERF1*, and *IbGH3* in sweet potato leaves (Figure 9), thereby promoting the growth of sweet potato seedlings.

## 4. Discussion

Endophytes are considered environmentally friendly microorganisms with a variety of biological activities [43,44]. Endophytic bacteria are closely related to plant development and nutrient acquisition; they reside in plant tissues and are an indispensable part of the plant microecosystem [45,46]. MEPW12 exhibited beneficial plant growth-promoting properties through the synthesis of siderophore, IAA and ACC deaminase, and CMC cellulase as well as its phosphorus-solubilizing and nitrogen-fixing abilities. Notably, IAA could indirectly promote root development while promoting cell elongation, thus significantly promoting overall plant development [47,48]. In addition, microbial phosphorus solubilization is essential to facilitate the plant uptake of nutrients from the natural environment [49,50], and nitrogen fixation combined with ACC deaminase activity provides an additional nutrition-beneficial nitrogen source for the host [51,52,53]. The production of CMC cellulase indicated the potential of MEPW12 to utilize lignocellulose [54]. The ability to produce siderophore proved that MEPW12 could promote plant growth by chelating Fe^3+^ [55]. The increased content of chlorophyll means that after inoculation with MEPW12, the photosynthesis of sweet potatoes was enhanced, thus accelerating growth [34].

Whole genome sequencing allows the rapid identification of genes associated with certain traits or phenotypes, such as from annotations in the genome of *Bacillus* sp. to genes or gene clusters associated with promoting plant growth and biocontrol activity. In this study, it was found that strain MEPW12 contained genes related to IAA synthesis, phosphate solubilization and uptake, nitrogen fixation, and siderophore production, which were consistent with others [56]. In addition, *acoA*, *acoB*, and *acoR*, key genes of Acetoin catabolism, which are known to promote plant growth, were also found in strain MEPW12 [57]. The presence of these genes indicates the potential of strain MEPW12 in promoting plant growth. Comparative genomes showed that the nucleotide sequences of MEPW12 were highly similar to those of other model strains, but there were also genome rearrangements such as turnover and translocation. It also has unique gene clusters related to metabolism, cellular processes and signaling, information storage, and processing. According to the results of the pan-genome analysis, it can be seen that the pan-genome of *Bacillus* sp. is open, which means that it can continuously obtain new genetic information from the external environment and has strong environmental adaptability, which is consistent with the results of other studies [58,59]. Microbial environmental adaptability is a core survival ability formed during the long-term evolution of microorganisms. This property not only enables them to survive in extreme or complex environments but also has multiple important values in ecosystems.

The application of growth-promoting endophytes can enhance the nutrient uptake of hosts, promote plant growth, enhance plant stress resistance, improve the soil environment, and regulate plant metabolism [3]. Wang et al. isolated a *Bacillus subtilis* strain GUCC4 from passion fruit, which had multiple activities such as IAA production, phosphorus solubilization, nitrogen fixation, and so on. It could significantly promote the growth of passion fruit seedlings and showed control effects on the pathogen *Nigrospora sphaerica* [29], which is similar to the function of MEPW12 in this study. Pitiwittayakul et al. also isolated a number of endophytic bacteria from sugarcane with the ability to promote plant growth and effectively control the disease caused by *Fusarium moniliforme* [60]. Tashan et al. isolated four root endophytic bacteria from alfalfa and chickpea roots grown in arsenic (As)-contaminated soil and found that they contribute to host growth in As-stressed soil [61]. Other researchers used endophytic bacteria on Oil Palm (*Elaeis guineensis*) and found that endophytic bacteria could induce an increase in vitamin B1 [62]. The above research implies that the study of endophytic bacteria can have numerous beneficial effects on the host.

Through gene expression quantification, we found that MEPW12 inoculation induced higher expression of *IbGH3*, *IbIAA1*, *IbERF1*, and other genes in sweet potatoes. Studies have shown that GH3 proteins play an important role in plant growth and development by regulating the homeostasis of auxin in plants [63,64]. The amide synthase encoded by the *GH3* gene catalyzes the binding of phytohormones such as auxin (IAA), jasmonate, and salicylic acid to amino acids, regulates the concentration of phytohormones, and is induced by exogenous phytohormones to regulate plant growth and development and stress response [64,65]. The Aux/IAA family plays a central role in the early auxin response and regulates the whole process of auxin signaling [66]. Aux/IAA proteins have been confirmed to be negative regulators of the auxin response and similarly induced expression by exogenous auxin [67], so it can be proved that MEPW12 can regulate the growth of sweet potatoes by producing plant auxin. Ethylene response factors (ERFs) are involved in plant growth and biotic/abiotic stress responses [68]. ERFs promote root and height growth, as well as respond to environmental stresses [69]. The high expression of ERF1 induced by MEPW12 may also be a potential mechanism for promoting the growth of sweet potatoes.

Microbial improvement in agroecology is an important environmental strategy [70,71]. The application of endophytic bacteria in the preparation of microbial agents can efficaciously enhance crop quality, ameliorate agricultural ecosystems, and remediate soil pollution [72]. Simultaneously, exploring the interaction mechanism between endophytes and their hosts, making full use of microbial resources, and finding functionally dominant strains in complex and extreme environments are of great significance for crop production, breeding, and sustainable agriculture.

## 5. Conclusions

A total of 12 endophytic bacteria were isolated from sweet potatoes in this study. Through functional identification and screening, it was found that *Bacillus amyloliquefaciens* strain MEPW12 had activities of phosphorus solubilization, nitrogen fixation, IAA production, sidersiderin production, cellulase production, and ACC deaminase production, which could promote the growth and chlorophyll content of sweet potato seedlings. Meanwhile, MEPW12 can inhibit the growth of pathogenic fungi in sweet potatoes and can use a variety of carbon sources and salt for growth. It can also grow in extreme environments of high salt and weak acidity. Whole genome sequencing revealed that strain MEPW12 not only contained multiple plant growth-promoting gene clusters but also had strong metabolite production potential. Comparative genomics and pangenome analyses showed that MEPW12 was broadly similar to the genomes of other Bacillus genera, and that the pangenome of *Bacillus* sp. is open to continuous acquisition of new genetic information from the external environment. Inoculation of MEPW12 into rhizosphere soil promoted the growth of sweet potato seedlings by inducing the expression of the genes IbGH3.10, IbIAA1, and IbERF1. In summary, *Bacillus amyloliquefaciens* strain MEPW12 is a plant growth-promoting bacterium with multiple functions, and this study provides a potential microbial resource for the sustainable development of agriculture.

## Figures and Tables

**Table 1:** Functional statistics of endophytes (‘+’ means functional; ‘−’ means no function).

Strain	FN	SP	CMC	ACC	Siderophore	IAA Concentration (mg/L, Average)
MEPW1	+	−	−	−	−	20.56
MEPW2	+	−	−	+	+	13.32
MEPW3	+	−	−	+	+	12.75
MEPW4	−	−	+	−	−	10.82
MEPW5	+	−	+	+	+	5.29
MEPW6	+	−	+	+	+	7.54
MEPW7	+	−	−	−	−	15.12
MEPW8	+	−	−	+	+	16.91
MEPW9	+	−	+	+	+	13.99
MEPW10	+	−	+	+	−	12.22
MEPW11	−	−	−	+	+	10.97
MEPW12	+	+	+	+	+	13.94

**Table 2:** Physiological and biochemical identification of MEPW12 (‘+’ means positive; ‘−’ means negative).

Functions	Results
V–P	+
Citrate	+
Propionate	−
D-xylose	+
L-arabinose	+
D-mannitol	−
Gelatin liquefaction	+
Growth with 7% NaCl	+
Growth with pH5.7	+
Nitrate reduction	+
Starch hydrolysis	+
Anaerobic growth	+

**Table 3:** Genes related to plant growth.

PGP Traits	Genes
IAA	trpA trpB trpC trpD trpE trpF
Siderophore	fepC ftsY ftsX ftsE
Nitrogen generation	glnA glnN glnH glnP glnM glnT glnK glnL nasB nasD nasE gltB gltD gltX
Phosphate solubilization and uptake	pstA pstB pstC pstS phnP phoD phoB1 phoP phoR ugpQ

**Table 4:** CAZy database annotations for strain MEPW12.

Class	Num	Descrition
GHs	40	Glycoside Hydrolases
GTs	35	Glycosyltransferases
PLs	3	Polysaccharide Lyases
CEs	17	Carbohydrate Esterases
AAs	6	Auxiliary Activities
CBMs	4	Carbohydrate-Binding Modules

**Table 5:** Prediction of secondary metabolic gene clusters using the antiSMASH database.

Region	Type	From	To	Most Similar Known Cluster	Compound	Similarity
Region1	NRPS	304,470	369,877	NRP:Lipopeptide	surfactin	78%
Region2	thiopeptide, LAP	585,389	614,502	Polyketide	kijanimicin	4%
Region3	RRE-containing, LAP	697,719	720,896	RiPP:LAP	plantazolicin	91%
Region4	PKS-like	925,178	966,422	Saccharide	butirosin A/butirosin B	7%
Region5	terpene	1,048,459	1,069,199	-	-	-
Region6	transAT-PKS	1,367,308	1,455,520	Polyketide	macrolactin H	100%
Region7	transAT-PKS, T3PKS, NRPS	1,674,324	1,784,435	Polyketide + NRP	bacillaene	100%
Region8	NRPS, transAT-PKS, betalactone	1,841,080	1,978,908	NRP	fengycin	100%
Region9	terpene	2,001,477	2,023,360	-	-	-
Region10	T3PKS	2,096,195	2,137,295	-	-	-
Region11	transAT-PKS	2,294,633	2,400,811	Polyketide	difficidin	100%
Region12	NRP-metallophore	3,030,529	3,082,321	NRP	bacillibactin	100%
Region13	other	3,599,564	3,640,982	Other	bacilysin	100%

## Data Availability

All data supporting the findings of this study are available within the paper and its Supplementary Information. The datasets generated and/or analyzed during the current study are available in the NCBI (National Center for Biotechnology Information) repository. The 16S rRNA sequence of *Bacillus amyloliquefaciens* strain MEPW12 has been uploaded to NCBI genbank (https://www.ncbi.nlm.nih.gov/genbank/) (Accession: PQ533237.1); Genomic data for *Bacillus amyloliquefaciens* strain MEPW12 are available from the NCBI database (https://www.ncbi.nlm.nih.gov/) (Accession number: PRJNA1191074); Transcriptome data of sweet potato (Xuzishu8) can be obtained from NCBI database (https://www.ncbi.nlm.nih.gov/) (Accession number: PRJNA1126169).

## Supplementary Materials

The following supporting information can be downloaded at https://www.mdpi.com/article/10.3390/microorganisms13061322/s1: Supplementary Figure S1. Endophytic bacteria isolated from sweet potato; Supplementary Table S1. Primers for qRT-PCR.

## Abbreviations

ACC	1-aminocyclopropane-1-carboxylic
IAA	Indole-3-acetic acid
CMC	Carboxymethyl cellulose
CAS	Chrome Azurol S
NF	Nitrogen fixation
SP	Solubilization of phosphate
CK	Control Check
qPCR	Quantitative Real-time Polymerase Chain Reaction
qRT-PCR	Quantitative Reverse Transcription Polymerase Chain Reaction
MYC2	Myelocytomatosis proteins 2
IAA1	Indole acetic acid 1
ERF1	Ethylene responsive factor 1
GH3.10	Gretchen Hage 3.10
JAZ6	Jasmonate ZIM-domain 6
PYL-2	Pyrabactin resistance 1-like 2
AUX1	Auxin 1
PR-1	Plant pathogenesis-related protein 1
EIN3	Ethylene-insensitive 3
