# Supplementary figures and images for: Genome-Driven Functional Validation of Bacillus amyloliquefaciens Strain MEPW12: A Multifunctional Endophyte for Sustainable Sweet Potato Cultivation
Source: Microorganisms. 2025 Jun 6;13(6):1322. doi: 10.3390/microorganisms13061322 (PMC12195239; doi:10.3390/microorganisms13061322)

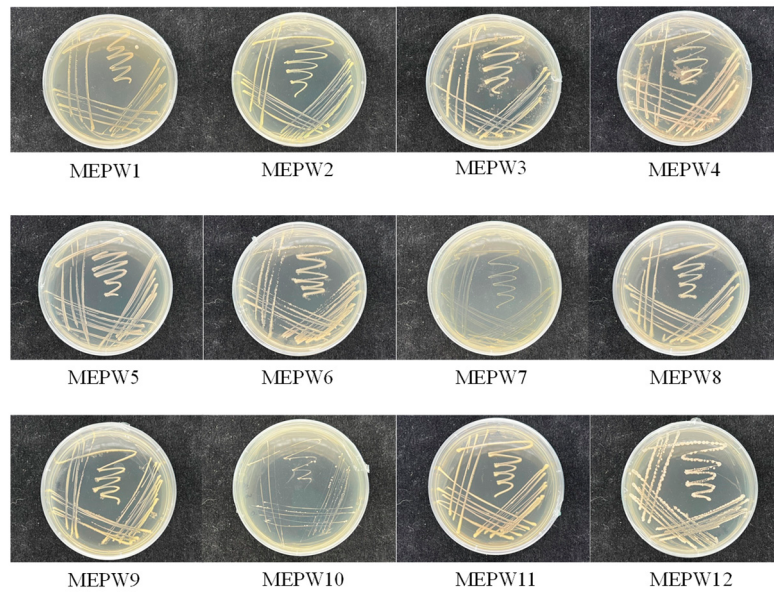

Supplementary Figure S1. Endophytic bacteria isolated from sweet potato

Supplement: Supplementary file 1 [file microorganisms-13-01322-s001.zip › Supplementary Figures.pdf]
